# Disclosure of Sexual Victimization: Effects of Invalidation and Shame on Re-Disclosure

**DOI:** 10.1177/08862605231155122

**Published:** 2023-02-20

**Authors:** Ashley K. H. Catton, Martin J. Dorahy, Kumar Yogeeswaran

**Affiliations:** 1 University of Canterbury, Christchurch, New Zealand

**Keywords:** sexual abuse, child abuse, anything related to child abuse, treatment/intervention

## Abstract

Research on disclosure of sexual victimization has consistently demonstrated that the act of disclosure and the disclosure recipient have a synergistic effect in facilitating either positive or negative post-assault outcomes. While negative judgments such as victim blame have been argued to serve a silencing function, experimental investigations of this claim are lacking. The current study investigated whether invalidating feedback in response to self-disclosure of a personally distressing event produced feelings of shame, and whether shame influenced subsequent decisions around re-disclosure. Feedback type (validating, invalidating, no feedback) was manipulated in a sample of 142 college students. Results partially supported the hypothesis that shame resulted from invalidation, however shame was better predicted by individual perceptions of invalidation than the experimental manipulation. Although few participants opted to make changes to the content of their narrative for re-disclosure, those who did had higher levels of state shame. Results suggest that shame may be the affective mechanism by which invalidating judgments silence victims of sexual violence. The present study also supports the distinction previously made between Restore and Protect motivations in managing this shame. This study provides experimental support for the notion that an aversion to being shamed, communicated via an individual’s perception of emotional invalidation, features in judgments of re-disclosure. Perceptions of invalidation, however, vary individually. Professionals working with victims of sexual violence should be mindful of the importance of shame attenuation in facilitating and encouraging disclosure.

## Disclosure of Sexual Victimization: Effects of Invalidation and Shame on Disclosure Inhibition

The ubiquity of victimization from sexual violence is an urgent problem to be addressed. Although surveys measuring the frequency and nature of sexual victimization vary in their methodology, international studies of 12-month incidence rates estimate that up to 59.2% of women and 55.5% of men are victims of sexual violence (Dworkin et al., 2021). The negative sequelae of sexual victimization are vast, including the acquisition of sexually transmitted infections, anxiety, depression, post-traumatic stress, dissociation, and self-blame (Aiken & Griner, 2022; Oshodi et al., 2020; Stermac et al., 2014; Ullman et al., 2007). While disclosure of sexual victimization is an important step towards recovery, the mere act of disclosing is not sufficient to ensure positive outcomes. For instance, it is argued that the first person a victim discloses to has a greater impact on the victim’s wellbeing than all subsequent disclosure recipients (Bonnan-White et al., 2018). Further, a victim’s decision to approach formal service providers is often preceded by disclosure to a close confidante (Barrios et al., 2022; Bonnan-White et al., 2018; Halstead et al., 2017; McElvaney et al., 2020). Indeed, victims may approach formal service providers such as counsellors or clinical psychologists for disclosure because of their perceived professional values of trust, confidentiality, and lack of judgment (Chouliara et al., 2011; McGregor et al., 2010). This suggests that the act of disclosure and the response by the disclosure recipient have a synergistic effect in facilitating recovery.

Researchers have identified distinct disclosure patterns among victims, capturing differences between timing, motivating reasons, and choice of disclosure recipient (Ahrens et al., 2010; Bicanic et al., 2015; Collings et al., 2005). Such patterns are defined in part by the salience of different barriers to disclosure. One common interpersonal barrier is the anticipation of negative social reactions (Carson et al., 2020; Sivagurunathan et al., 2019; Weiss, 2010). Negative social reactions are responses to disclosure that provide unhelpful or harmful feedback, and include expressions of disbelief, and stigmatizing responses such as victim blame (Catton & Dorahy, 2022; Iles et al., 2021; Wager et al., 2021). Such responses may produce distress if they violate the victim’s expected reaction from a disclosure recipient (Dworkin et al., 2019). Reactions can include taking control or attention away from victims and their situation or stigmatizing and blaming them for their behavior (Ullman, 2000). Stigmatizing responses can invalidate the victim’s (a) experiences, (b) interpretation of their experiences, or (c) their decision to disclose. Relatedly, negative reactions to rape disclosure have been argued to serve a silencing function by reinforcing extant self-blame and uncertainty about whether the victim’s experience qualifies as rape (Ahrens, 2006).

Invalidation informs the victim that their interpretation of their own experience is wrong while also labeling their reaction as socially unacceptable (Linehan, 1993). Rather than ameliorating distress and providing a narrative that places the victim’s interpretation and experience within an accepted social context, invalidating responses maintain the disunity between the victim’s experience and an accepted social expression in which to channel that experience. Indeed, invalidating feedback has been associated with increased physiological arousal and negative affect (Greville-Harris et al., 2016; Shenk & Fruzzetti, 2011; Weber & Herr, 2019). Invalidation has also been associated with avoidance behavior. For instance, Greville-Harris et al. (2016) invalidated participants and found that 80% declined to participate in further research. Seemingly strong affective underpinnings motivate post-invalidation behavior. Weber and Herr (2019) found a moderate effect for negative affect associated with invalidation, but no significant effect for the specific emotions of guilt or sadness. Thus, the immediate affective product of invalidation remains to be identified. Invalidation communicates what qualities and behaviors are socially unacceptable and thus stigmatized. Stigma confers a threat of shame that imposes social control by enforcing in-group boundaries (Lewis, 2003). To bear stigma is to have one’s social image tainted or discounted due to having some attribute that is socially undesirable (Goffman, 1968). Thus, alongside challenging victims’ interpretation of their own experience, invalidating feedback may also communicate that the act of disclosure has violated a social norm, and the victim is punitively shamed for their transgression.

Theories of shame often refer to aspects of the social self, specifically its preservation and position on a social hierarchy (Dickerson et al., 2009; Dickerson & Kemeny, 2004; Gilbert, 1997). Shame is associated with global, stable, internal attributions (Tracy & Robins, 2006; Van Vliet, 2009). Hence, shame arises in situations where a social transgression is perceived to reflect something permanent about one’s character. In line with this, research has found strong associations between shame and attributions of self-blame, particularly characterological self-blame (Tilghman-Osborne et al., 2008; Van Vliet, 2009). As such, shame is associated with a conscious belief that the self is flawed or defective in the eyes of others. Thus, state shame is an affective state stemming from an undesired shift in one’s social standing and is experientially and qualitatively similar to feelings of humiliation and degradation (Gilbert, 1997). Hence, elicitors of shame entail a threat to the social self—where an individual’s social identity may be discredited (Kemeny et al., 2004; Macdonald, 1998). Budden (2009) outlines two classes of social threat associated with shame: domination/subjugation, and acute norm violation. As invalidation signals norm violation, it follows that invalidating feedback ought to produce shame. In addition to shame in the presence of social threat, victims of interpersonal violence, such as sexual assault, are likely to feel shame given the physical dominance such assaults entail. Consistent with this, shame has been reported by victims of sexual violence. For example, DeCou et al. (2019) reported a small positive correlation between assault-related shame and assault severity. Drawing on national crime victimization data, Weiss (2010) found that victims reported shame-related phenomena such as feelings of humiliation, attributions of self-blame, and fear of public scrutiny. More generally, individuals with posttraumatic stress disorder have been shown to be hyper-sensitive to social threat (Stevens & Jovanovic, 2019). As a result, it is likely that victims’ hypervigilance to being shamed is sufficient to inhibit disclosure.

While the prototypical behavioral response to shame is submission (e.g., Gilbert, 1998), there are individual differences in how shame is managed. For instance, shame is often associated with avoidance tendencies (Schmader & Lickel, 2006). However, shame has also been shown to motivate approach behaviors to restore the positive reputation of an individual’s social identity, and this may be preferable to avoidance if there is minimal risk of further damage (de Hooge et al., 2010, 2011). However, the preference for approach or avoidance may also depend upon the individual’s willingness to internalize shame. Nathanson (1992) argues that internalized shame refers to an acceptance of the flawed self, whereas externalized shame is a deflection and rejection of the shame message resulting in the aggressive maintenance of one’s positive social identity, at the expense of others.

While there is a dearth of research on shame coping behavior, other work may suggest which factors could be implicated. For instance, a study investigating the connection between non-disclosure and self-blame explained this association as a function of self-esteem protection (avoidance of exposure), and preservation of interpersonal relationships by avoiding disapproval and/or rejection (Finkenauer & Rime, 1998). Relatedly, interpersonal sensitivity has been associated with shame internalization in work by Nystrom et al. (2018), where they explained that shame may be internalized to preserve personal relationships. Thus, it is likely that the prioritization of an individual’s social relations relative to the maintenance of their social identity is an important determinant in how shame is managed. Regarding disclosure, this would result in determining whether receiving validation was more important than maintaining or restoring positive social relations after receiving an invalidating response. If positive social relations were prioritized, norm conformity may stem from shame internalization, where either an acceptable reformulation of the disclosed narrative occurs, or an aversion to re-disclosure altogether develops. Conversely, avoidance of disclosure might also be associated with a primary motive to maintain a positive social image, suggesting that disclosure would not be a viable option at any stage. However, these theoretical possibilities remain to be tested.

To date, there has been no experimental research examining if invalidating feedback to disclosure of a shame-related event, such as sexual assault, produces feelings of shame. In addition, it is not yet known if shame elicited from invalidation causes an aversion to re-disclosure, or a desire to reformulate the disclosure narrative to conform with perceived social norms. It is also unclear if individual differences in coping with shame results in distinct motivations for re-disclosure. The present research addresses these aims with the following hypotheses:

Hypothesis 1: There would be a significant difference in levels of state shame depending on whether individuals received validating or invalidating feedback to self-disclosure, such that those who received invalidating feedback would exhibit the highest levels, and those with validating feedback the lowest.
Hypothesis 2: Those who received validating feedback would be more likely to re-disclose to a new disclosure recipient, than those who received no feedback or invalidating feedback——the latter of which would have the weakest association with this outcome.
Hypothesis 3: Those receiving invalidating feedback would be more likely to amend their narrative for re-disclosure.
Hypothesis 4: The association between invalidation and amendment to the disclosed narrative in the invalidating condition would be driven by changes in state shame.

Due to a lack of previous empirical and theoretical work on the topic, there was no expectation about direction and strength of the relationships between disclosure choices around changes to content or recipient, the motivations underlying those choices, and the presence of expectancy violations.

## Method

### Participants

One hundred and forty-two psychology undergraduate students from a New Zealand university were recruited as a convenience sample, and all completed the study. They ranged in age from 16 to 52 years (Mean [*M*] = 21.92; Standard Deviations [*SD*] = 6.46). One hundred and six were women (74.6%), 33 were men (23.2%), and three identified as “other” (2.1%). Most participants (82.4%, *n* = 117) identified as New Zealand European, with a minority identifying as Māori, Pacific Islander, Chinese, Japanese, or other ethnicities. Participants received course credit for their participation in the study. The study was approved by the University’s Human Ethics Committee.

### Materials

#### Self-report measures

The demographic questionnaire assessed participants’ age, gender, ethnicity, level of education, mental health history, and relationship status.

##### The Compass of Shame Scale – modified

The Compass of Shame Scale (COSS) is a self-report questionnaire measuring the shame-coping styles of Avoidance, Attack-Self, Withdrawal, and Attack-Other (Elison et al., 2006). An approach dimension was developed for inclusion with the COSS to produce the Compass of Shame Scale – modified (COSS-m) (Wu, 2020). The revised scale includes 60 items, across 12 scenarios assessing each of the five coping styles. Participants rate the frequency with which they find themselves using each coping style in each scenario (e.g., “At times when I am unhappy with how I look. . . [a behavior based on each of the five coping styles is then presented]”). Responses range from 0 (*never*) to 4 (*almost always*). Higher scores indicate an increased tendency to adopt that coping style. The COSS-m has good psychometric properties, specifically construct and face validity (Wu, 2020) and in this study had good internal consistency (Cronbach’s α = .90).

##### The Experience of Shame – behavioral subscale

The Experience of Shame – behavioral subscale (ESS-b) is a 9-item self-report subscale of the ESS measuring levels of shame associated with an individual’s own behavior (Andrews et al., 2002). Items ask respondents whether feelings of behavioral shame have occurred to them during the past year (e.g., “Have you avoided people who have seen you fail?”). Responses range from 1 (*not at all*) to 4 (*very much*). Higher scores reflect greater behavior-related shame-proneness, with a maximum score of 36. The ESS-b has good psychometric properties such as internal consistency and test-retest reliability (Andrews et al., 2002) and in this study had a Cronbach’s α of .86, indicating good internal consistency. The ESS-b was used to prime participants for memories of behavioral shame.

##### Perceived Emotional Invalidation Inventory

The Perceived Emotional Invalidation Inventory (PEII) is a 10-item self-report measure gauging respondents’ perception of emotional invalidation at the present moment (Elzy & Karver, 2018). Items ask respondents whether they feel their emotions have been invalidated by another party (e.g., “I felt ignored when I shared my feelings”). Responses range from 1 (*strongly disagree*) to 5 (*strongly agree*). Higher scores indicate higher levels of perceived invalidation, with a maximum score of 50. Several items were amended to refer to “the psychologist” as the source of feedback for the present study. The PEII has demonstrated high reliability and construct validity (Elzy & Karver, 2018) and in the current study had a Cronbach’s α of .94, reflecting good internal consistency.

##### Restore/Protect Scale

The Restore/Protect Scale (RPS) is a 10-item self-report scale measuring respondents’ tendency toward either reputational restoration or self-protection following an experience of shame (de Hooge et al., 2010). Items ask respondents to reflect on the reasons underlying decisions they have been presented with in the experimental setting (e.g., “Improve my self-image”). Responses range from 1 (*not at all*) to 7 (*very strongly*). Higher scores indicate higher motivational tendencies, with a maximum score of 35 for each tendency. The RPS has demonstrated sound construct validity (de Hooge et al., 2010) and in the current study had good internal consistency with a Cronbach’s α of .90 for the Restore scale, and .95 for the Protect scale.

##### The State Shame Scale

The State Shame Scale (SSS) is a 5-item self-report scale measuring feelings of shame as they are felt in the present moment and is a subscale of the State Shame and Guilt Scale (Marschall et al., 1994). Items ask respondents whether feelings of shame are being felt in the moment (e.g., “I want to sink into the floor and disappear”). Responses range from 1 (*Not feeling this way at all*) to 5 (*Feeling this way very strongly*). Higher scores indicate greater levels of state shame. The SSS has sound construct validity (Ghatavi et al., 2002) and had a Cronbach’s α of .90 in this study, indicating good internal consistency.

##### Expectancy violation

Two questions were asked of participants in the Validation and Invalidation conditions that gauged how much they believed their received feedback was typical of what a clinical psychologist would offer, and how much they expected that feedback. Respondents rated their agreement from 0 (*not typical at all*) to 10 (*very typical*).

#### Disclosure task

After completing and being guided by the questions within the ESS-b, participants were asked to disclose an instance of behavioral shame but were cautioned not to disclose something they found personally traumatizing. To aid this, they were presented with a visual scale from 0 (*no distress*) to 10 (*extremely distressing*) and asked to recall experiences within the range of 5 to 8 on the scale. They typed out a paragraph detailing what happened and how they felt about the event before clicking on a button to submit their story to a clinical psychologist who was on standby to evaluate it. A psychologist was chosen as the disclosure recipient because clinical psychologists are central to providing therapy and support to victims of sexual violence. Moreover, clinical psychologists are considered to have many qualities important for disclosure recipients such as confidentiality, lack of judgment, and a willingness to listen (Chouliara et al., 2011). However, all responses from the psychologist were presented automatically according to a timed programmed script. After being presented with a “wait” screen for 30 s and depending on the assigned condition, they received either validating feedback, invalidating feedback, or were given an error message indicating that no feedback was provided within the timeframe expected. The exact wording of these is available online at https://osf.io/xth8r/. Participants were then asked whether they wanted to amend their narrative before continuing, and whether they wanted to redisclose to the same psychologist they received feedback from, or a different psychologist. Two measures stemmed from this task: Disclosure Content, and Disclosure Recipient.

### Procedure

The study was completed with the researcher using an online survey platform on a PC computer in a lab setting. Participants were recruited within the psychology department to take part in a study entitled “Social Sharing of Emotional Experiences.” Upon completing the written consent process, they were informed of the nature of the tasks involved. Participants completed the demographic questionnaire, and any participant who indicated a diagnosis of a trauma-related disorder was automatically excluded from the invalidation condition. Participants then completed the COSS-m followed by the ESS-b. Next, they were given the disclosure task where they were asked to disclose an event of behavioral shame in writing to a clinical psychologist who was on standby to read their narrative. Participants were then instructed to complete the SSS and PEII and were then presented with the two disclosure choices. After indicating their choices, all participants received validating feedback after a 30 s delay. They were then administered the RPS and asked how believable they perceived the psychologist’s responses to be before being debriefed. The debrief phase consisted of distress alleviation if necessary, asking participants about their experience of the study, and whether they had any strong experiences during their participation that was not captured in any of the state or trait measures they completed.

### Design and Data Analysis

The study was a between-subjects single factor design. The independent variable was disclosure feedback type: validation, invalidation, or no feedback. The main dependent variables were (a) State shame, (b) Disclosure content, (c) Disclosure recipient, and (d) Expectancy violation. Perceived Invalidation was intended as a manipulation check. SPSS v23 (IBM Corp, 2015) was used to perform statistical analysis.

## Results

Data, including additional tables, are available at: https://osf.io/xth8r/. In examining the distribution of each scale, only State Shame was skewed at 1.28 (*SE* = 0.20), and the skew remained after attempting an inverse transformation, making this data suitable only for non-parametric analyses. Table 1 shows the means and *SD* for the rating scales. Correlation coefficients were calculated for all state measures as shown in Table 2 and illustrate a large positive correlation between Perceived Invalidation and State Shame. A one-way ANOVA examining levels of Perceived Invalidation between the Validation and Invalidation conditions found a significant large effect for feedback type, *F* (1,96) = 58.20, *p* < .001, *η*_p_^2^ = .38. This was repeated for both Perceptions of Expectancy and Typicality, finding significant moderate effects for Perceived Typicality (*F* [1,96] = 20.26, *p* < .001, *η*_p_^2^ = .17), and Perceived Expectancy (*F* [1,93] = 11.63, *p* = .001, *η*_p_^2^ = .11). This indicates that participants in the Invalidation condition perceived their feedback was more invalidating than those in the Validation condition, and this feedback was not believed to be typical of what a clinical psychologist would offer nor was it expected.

**Table 1.** Means (*M*), Standard Deviations (*SD*), and Number of Participants (*n*) for Self-Report Measures.

Scale	*n*	Minimum	Maximum	*M*	*SD*
Compass of Shame: Approach	142	14	47	28.96	5.99
Compass of Shame: Attack Other	142	0	34	12.67	7.01
Compass of Shame: Attack Self	142	4	48	29.38	9.26
Compass of Shame: Avoidance	142	9	36	23.05	5.23
Compass of Shame: Withdraw	142	3	47	25.10	8.86
Experience of Shame: Behavioral Subscale	142	9	36	25.66	5.29
Perceived Expectancy of feedback	95	0	10	3.78	2.90
Perceived Invalidation Inventory	98	10	47	24.91	10.10
Perceived Typicality of feedback	98	0	10	4.07	3.12
Protect Motivation	142	5	35	13.13	8.29
Restore Motivation	142	5	35	16.04	7.67
State Shame Scale	142	5	23	8.52	4.38

**Table 2.** Correlations for State Measures.

Variable	*n*	1	2	3	4	5	6
1. Perceived Invalidation Inventory	98	—					
2. Perceived Expectancy of Feedback	95	−.42*	—				
3. Perceived Typicality of Feedback	98	−.51*	.79*	—			
4. Protect Motivation	142	.40*	−.07	−.02	—		
5. Restore Motivation	142	−.26*	.12	.12	.63*	—	
6. State Shame	142	.59*	−.15	−.12	.53*	.34*	—

* *p* < .01.

A Kruskal–Wallis test conducted for testing the first hypothesis that there would be differences in state shame by feedback type found a non-significant trend for differences in State Shame between conditions (see Supplemental Table at https://osf.io/xth8r/), *H*(2) = 4.88, *p* = .09. As this hypothesis specifically stated that Shame levels would be lower in the Validation condition compared to the Invalidation condition, post-hoc Mann-Whitney tests revealed a significant difference between State Shame in the Invalidation (mean rank of 55.81) than Validation (mean rank of 43.44) condition with a small effect; *U* (*N*_validation_ = 50, *N*_invalidation_ = 48) = 897.00, *z* = −2.21, *p* = .03, *r* = .22.

To investigate whether Feedback Type or Perceived Invalidation was more predictive of State Shame, a hierarchical linear regression was performed but the resulting model violated assumptions of normality, even when an inverse transformation of State Shame was attempted. To recover the data for this analysis, a new categorical variable of High/Low Shame was created from a median split of the SSS. A hierarchical logistic regression was then performed, with Perceived Invalidation (Exp[*B*] = 1.14, *p* < .001) as the first step in a model, *Χ*^2^(1) = 30.20, *p* < .001, explaining 39% of the variance in Shame, Nagelkerke *R*^2^ = .39. The addition of Feedback Type in the second step was not a significant predictor (Exp[*B*] = .30, *p* = .06) and the resulting model, *Χ*^2^(2) = 34.19, *p* < .001, did not increase the proportion of variance accounted for (Nagelkerke *R*^2^ = .39). This suggests that individual beliefs about the nature of the feedback received were more predictive of state shame than whether that feedback was explicitly validating or invalidating.

Given the small effect for Feedback Type on State Shame, and the large effect for Feedback Type on Perceived Invalidation, it may be that the relationship between Feedback Type and State Shame is mediated by Perceived Invalidation. The Hayes (2017) PROCESS macro for SPSS was used to conduct a simple mediation analysis with High/Low Shame as the outcome, Feedback Type as the predictor, and Perceived Invalidation as the mediator, with 5,000 bootstrap samples generated. While a direct effect for Feedback Type on High/Low Shame approached significance (*b* = −1.21, *SE* = 0.64, *p* = .06, 95% CI [–2.5, 0.04]), there was greater support for an indirect effect via Perceived Invalidation as the bootstrap confidence interval estimates did not include zero; *b* = 2.15, *SE* = 0.55, [1.35, 3.52]. This further supports the conclusion that the effect of feedback type on feelings of shame is explained by individual perceptions of invalidation. However, upon comparing results for the direct effect of Feedback Type on High/Low Shame, and the relationship between Feedback Type and Perceived Invalidation, the direction of the relationship in both instances differed. Specifically, the direct effect coefficient was negative, yet the coefficient for Feedback Type on Perceived Invalidation was positive (*b* = 12.35, *SE* = 1.62, *p* < .001, [9.14, 15.56]). As the direct effect merely approached significance, this suggests some participants in the validation condition felt shame that was not explained by perceptions of invalidation.

The range of experiences disclosed (from minor embarrassment to highly traumatizing experiences) and the amount of information conveyed within each disclosure suggested that further understanding may be gained from factors that contribute to both participants’ perceptions of invalidation, and subsequent disclosure choices. Thus, each narrative was coded by the experimenter (a clinical psychology doctoral student) for word length, percentage of narrative that references the self (e.g., possessives and pronouns), percentage of narrative referencing the participants experience of an emotion (e.g., “I felt ashamed”), and intensity of the amount of distress likely experienced by participants in the situation they disclosed using the same scale that participants were presented with. References to emotion and self have been used in other narrative assessments and have been found to be important in understanding an individual’s self-concept (Dost-Gözkan & Küntay, 2014). Inter-rater reliability was calculated after a second rater evaluated a random sample of 50 narratives, with weighted kappa values suggesting almost perfect agreement for the number of references to self (κ = .88, *p* < .001), and moderate agreement for number of references to emotion (κ = .59, *p* < .001) using guidelines by Landis and Koch (1977). Further, an Intraclass Correlation Coefficient was calculated for distress ratings (ICC = .88, *p* < .001), resulting in excellent agreement per Cicchetti (1994) guidelines.

A correlation between State Shame and Distress illustrated a significant positive association (*r* = .23, *p* = .005), indicating that the severity of the events disclosed related to feelings of shame during participation in the study. A Supplemental Table showing the distribution of these ad-hoc measures is available at https://osf.io/xth8r/. In addition, references to self positively correlated with Restore motivation (*r* = .21, *p* = .01), and references to emotion positively correlated with Protect motivation (*r* = .24, *p* = .005), indicating that distinct types of personalization of the disclosed narrative were associated with different post-feedback motivations. By contrast, overall narrative length negatively correlated with Restore motivation (*r* = −.19, *p* = .02). To examine whether any of these ad-hoc measures predicted Perceived Invalidation, a backwards stepwise linear regression was performed that included these new measures alongside Feedback Type and Perceptions of Typicality and Expectancy. The resulting model explained 45% of the variance, *F* (2, 92) = 37.85, *p* < .001, with Feedback Type (β = .48, *p* < .001), and Perceived Typicality (β = −.31, *p* < .001) making significant unique contributions. This indicates that individual perceptions of invalidation are informed by the feedback received and beliefs about its typicality.

To test the second hypothesis, that recipients of validating feedback would be more likely to approach a new disclosure recipient for re-disclosure, a Chi-Square test assessing differences in changes to Disclosure Recipient between conditions showed a non-significant result, *Χ*^2^ (2, 142) = 3.98, *p* = .14. This test was repeated for the third hypothesis, that there would be a significant difference in changes to Disclosure Content between conditions. The result was not significant, *Χ*^2^ (2, 142) = 2.07, *p* = .36. The number of changes to Disclosure Recipient did not differ markedly between conditions, and few participants (*n* = 14) made changes to Disclosure Content (see Supplemental Table at https://osf.io/xth8r/). Half of the changes to Disclosure Content were in the Invalidation condition and tended to include the addition of information that justified their emotional experience (i.e., the inclusion of more reasons to explain the emotion they described experiencing). However, one participant re-disclosed a different event that was more extreme. Changes in the Validation condition involved adding more contextual detail or emotion, suggesting that validation encouraged some participants to be more forthcoming about their experience. No discernible pattern was evident among changes in the No-Feedback condition.

Due to the nonsignificant result for the third hypothesis, the planned mediation analysis to test the fourth hypothesis that State Shame would mediate the difference between feedback type and changes to Disclosure Content, was not performed. However, a Mann-Whitney test was conducted to assess differences in State Shame using Disclosure Content as the grouping variable. This analysis was significant with a small effect; *U* (*N*_change_ = 14, *N*_leave_ = 128) = 572.00, *z* = −2.28, *p* = .02, *r* = .19, indicating that participants who changed their Disclosure Content had higher levels of State Shame than those who did not, regardless of Feedback Type.

Correlation coefficients between disclosure choices, motivation types, and trait measures are presented in Table 3. Given the large correlation between both motivation types (Restore, Protect), a backwards stepwise binary logistic regression was performed with both motivation types examined as predictors of change to Disclosure Content. This resulted in a model consisting of only Restore Motivation as a significant predictor (Exp[*B*] = .91, *p* = .01) of Disclosure Content, *Χ*^2^(1) = 7.03, *p* = .01, that explained 10% of the variance (Nagelkerke *R*^2^ = .10). Thus, Restore Motivation positively predicted disclosure change. However, upon comparing levels of Restore and Protect motivations for those who changed content, Restore was consistently high across all conditions (*M* = 21.53, *SD* = 6.65), whereas Protect was more variable (*M* = 18.07, *SD* = 9.73) due to it being much lower in the Validation (*M* = 11.67, *SD* = 7.02) and No-Feedback (*M* = 11.75, *SD* = 10.24) conditions than the Invalidation condition (*M* = 23.63, *SD* = 7.39). A one-way ANOVA found a significant effect for Protect motivation between conditions, *F* (2,12) = 4.00, *p* = .047, *η*_p_^2^ = .40, showing that among those who made changes to their narrative, invalidation elicited higher levels of Protect motivation than validation or no feedback. The same regression procedure was repeated for changes to Disclosure Recipient comparing the Withdraw and Attack Self scripts of the COSS, given their correlation with Disclosure Recipient, and only the Withdraw trait measure remained as a significant predictor (Exp[*B*] = 1.05, *p* = .03) in a model, *Χ*^2^(1) = 5.17, *p* = .02, which explained 5% of the variance; Nagelkerke *R*^2^ = .05. The direction of this relationship suggests that the Withdraw script predicts participants’ decision to approach a new recipient for re-disclosure.

**Table 3.** Correlations for Disclosure Choices, Motivation, and Trait Measures.

Variable	*n*	1	2	3	4	5	6	7	8	9	10
1. Changes to Disclosure Content	142	—									
2. Changes to Disclosure Recipient	142	.07	—								
3. Restore Motivation	142	−.22**	−.04	—							
4. Protect Motivation	142	−.18*	−.04	.63**	—						
5. Experience of Shame: Behavioral Subscale	142	−.09	.14	.09	.21*	—					
6. Compass of Shame: Attack Self	142	.10	−.17*	.07	.16	.69**	—				
7. Compass of Shame: Avoidance	142	.09	.06	.17*	.19*	.20*	.32**	—			
8. Compass of shame: Withdraw	142	−.06	.19*	−.02	.20*	.69**	.75**	.28**	—		
9. Compass of Shame: Attack Other	142	−.07	.11	.09	.20*	.39**	.40**	.42**	.46**	—	
10. Compass of Shame: Approach	142	−.03	.01	.33**	.17*	.02	.17*	.24**	−.15	−.14	—

* *p* < .05. ** *p* < .01.

## Discussion

This study tested four hypotheses. The first hypothesis was that there would be a difference in state shame across experimental conditions of differing feedback from a psychologist. Second, it was hypothesized that those who received validating feedback would be more likely to approach a new disclosure recipient for re-disclosure. Third, we hypothesized that those who received invalidating feedback would be more likely to amend their narrative for re-disclosure, and fourth, that this relationship would be mediated by state shame.

The finding that state shame did not differ overall between the three conditions, and only a small effect arose between the validating and invalidating conditions was unexpected. Rather, the relationship between invalidation and shame was better explained by individual perceptions of invalidation in the feedback received. This echoes the distinction in the disclosure literature between perceived and received feedback. Dworkin et al. (2019) describes received responses as those that are objectively observable whereas perceived responses are the individual appraisals of received responses. In practice, Dworkin et al. (2018) found that responses categorized as “positive” by researchers were received negatively by disclosers if (a) they violated the discloser’s expected response, (b) were unhelpful, or (c) made the individual feel uncomfortable—especially if they did not have a close relationship with the disclosure recipient. In addition, Elzy and Karver (2018) found greater variability among self-reported perceived invalidation scores when participants were invalidated, than when they received neutral feedback. Taken together, this suggests that individual differences in how feedback is perceived can vary to a large degree. Researchers and professionals dealing with victims of sexual violence must be mindful of the way in which their feedback may be interpreted differently within the population.

Regarding the present study, as participants understood they were disclosing to a psychologist, it is possible that the brief response given in the validation condition lacked the insight or specificity that might be expected from a psychologist and was perceived as invalidating. This suggests that invalidation may produce shame through either explicit expression or implicit impression. As an explicit expression of invalidation, this communicated to participants that their choice of a shame-related event to disclose was not sufficiently severe to justify disclosure as their distress was disproportionate to the events described. As psychologists may be regarded as authority figures on emotions, their feedback communicated that participants’ distress was not normal. By fostering disunity between subjective experience and an acceptable social expression of it, it is likely that shame resulted from the social threat of a discredited social identity from a perceived norm violation (Budden, 2009; Kemeny et al., 2004; Macdonald, 1998). In other words, explicit invalidating feedback conveys that the distress or shame participants expressed was disproportionately high and thus inappropriate relative to the situation participants described. By contrast, as an implicit impression, particularly for those in the validation condition, shame may have arisen from an attribution made by participants about the lack of depth or substance in the feedback received, that the psychologist did not invest enough time to compose a more considered response and this reflected something about themselves (e.g., “I’m not valuable enough for the psychologist to provide a more dedicated response”). While both the explanation and the distinction between explicit and implicit invalidation as elicitors of shame remain to be further explored, this interpretation suggests that a response designed to be validating can be perceived as unsympathetic. Indeed, dismissive reactions are argued to shut down discussion on the topic of sexual victimization, thus discouraging further disclosure (Scoglio et al., 2022).

The large effect of perceived invalidation between the validation and invalidation conditions did not result in a similarly large effect for state shame. This indicates that some participants who received invalidating feedback, and perceived it as such, did not report feeling shame. This may be due to the diverse nature of the subjective experiences disclosed. While participants were guided toward disclosing an intense but not extreme shame-related event, there was little control over what events were disclosed and how much personally meaningful information was included in the narrative. For instance, distinctions have been made between the breadth, length, and depth of disclosure, the inclusion of which are determined by multiple contextual factors (Omarzu, 2000). Given this, it follows that decisions around breadth and depth of disclosure are associated with how personally meaningful the disclosed narrative is, such that the narrative is a characteristic expression of the person disclosing it, thus increasing the risk of shame.

Similarly, results suggested that some participants may have reported feeling shame that was not explained by perceived invalidation. Given the relationship between state shame and the level of distress perceived to have been experienced in the disclosed events, it is possible that shame was either primed via the recollection of a distressing event, or disclosure of a more extreme event conferred greater shame-related exposure through the risk of negative evaluation. However, it is also possible that after being validated, maintaining unity between experience and expression, some participants were more forthcoming reporting their shame. Lepore et al. (2004) found that participants who were validated reported higher levels of distress the day after participating in the experiment than those who received feedback that “challenged” their disclosure. In this way, validation may encourage individuals to both experience and disclose their emotions more freely. This possibility, however, also implies that invalidated individuals are less likely to share their emotional experiences honestly and is another possible explanation for the lower levels of self-reported shame in the invalidation condition. Invalidation has been argued to communicate to the invalidated individual that their interpretation of their own experience is wrong (Linehan, 1993). This possibility is supported by recent findings that trait invalidation, the tendency to experience invalidating responses, is inversely associated with emotional expressiveness yet positively associated with emotional reactivity per self-report measures (Schreiber & Veilleux, 2022). Hence, future studies may benefit from directly investigating the role of validation and invalidation in affecting individuals’ willingness to be forthcoming and honest about their experiences, while exerting tighter control over the range of experiences being disclosed.

The lack of support for the second hypothesis, that participants whose disclosure was validated would be more likely to approach a new disclosure recipient for re-disclosure, was also surprising. It is unclear what might motivate individuals to re-disclose to the same recipient after receiving either validating or invalidating feedback, but this plausibly reflects a desire for more feedback than was originally provided. Regarding the decision to approach a new disclosure recipient, the presence of a small association between this choice and the Withdrawal response suggests that choosing a new disclosure recipient may have been motivated by a means of coping with shame. Specifically, the Withdrawal trait refers to a tendency to retreat from a shaming situation and is associated with escape-avoidance tendencies and interpersonal sensitivity (Elison et al., 2006; Nathanson, 1992). In this case, shame experienced from the first disclosure was, for some participants, an experience they did not wish to repeat and escaped the situation using the only other option immediately and obviously available to them. Thus, some participants may have avoided re-disclosure altogether if this choice was available.

While not directly supported, the third and fourth hypotheses that participants who had been invalidated were more likely to change the content of their narratives due to feeling more shame, were indirectly supported by post-hoc analyses. Participants who changed their narrative content felt more shame and were more likely to be motivated to restore their social image in the eyes of the original disclosure recipient. This echoes findings in the literature that non-disclosure and shame internalization are associated with a higher prioritization of social connections over self-assertion (Finkenauer & Rime, 1998; Nystrom et al., 2018). By perceiving disapproval or rejection in the feedback received, some participants were motivated to redeem themselves by amending the version of events originally expressed. However, very few participants opted to make changes, which limits the confidence with which this can be firmly concluded.

Taken together, these results support the notion that feedback perceived as invalidating after disclosing a personal event produces feelings of shame. Regarding previous findings showing that invalidation is associated with increased physiological arousal and general measures of negative affect, or reductions in positive affect (Benitez et al., 2020; Greville-Harris et al., 2016; Schreiber & Veilleux, 2022; Shenk & Fruzzetti, 2011; Weber & Herr, 2019), it is likely that shame was a contributing factor. Further, the finding by Greville-Harris et al. (2016) that invalidated participants were least inclined to participate in further research may be explained by shame-related avoidance. These results also support accounts that shame is associated with attempts to restore a damaged social identity if this can be done without further damage (de Hooge et al., 2010, 2011). For instance, some participants in the present study chose to amend their narrative for re-disclosure to the original recipient after being invalidated, whereas others opted to preserve their narrative and re-disclose to a new recipient. This may have resulted from a snap decision about which options were associated with the least risk of further invalidation and/or shame. Some models describe disclosure as a balancing act between subjective utility and risk (Omarzu, 2000), or visibility and concealment (Chaudoir & Fisher, 2010). In the present study, participants may have balanced the potential utility of social image restoration, motivating increased visibility, against the risk of further shame, motivating protection through concealment, the latter being more salient among those who were invalidated. However, it remains unclear whether or how shame inhibits further disclosure. Regardless, it is apparent that an aversion to being shamed is a salient factor in determining whether to re-disclose. This has important implications for professionals working with victims of sexual violence and speaks to the importance of attenuating shame in an effort to both encourage an initial disclosure and to avoid discouraging further re-disclosure.

While it has been suggested that negative outcomes associated with the receipt of negative responses to self-disclosure may be explained by expectancy violations (Dworkin et al., 2019), this was supported only by correlation in the present study. Participants endorsed lower levels of expectancy and typicality for invalidating than validating feedback, yet neither of these beliefs predicted shame. By contrast, perceived typicality of feedback was more predictive of perceived invalidation, the latter being a predictor of shame. As such, this at least supports the notion that cognitive factors, such as anticipatory beliefs and the evaluation of feedback received, may lead to maladaptive outcomes.

### Limitations and Future Directions

The data collection phase ended prematurely due to a COVID-19-related lockdown, resulting in a smaller sample size than anticipated. In addition, the feedback designed to be validating was perceived by some participants as invalidating, and this was likely due to participants expecting more personal feedback from a psychologist. This may be overcome by research designs with verbal disclosure to a confederate as has been the predominant approach (e.g., Elzy & Karver, 2018; Greville-Harris et al., 2016; Lepore et al., 2000, 2004;Shenk & Fruzzetti, 2011) or written disclosure to an anonymous “peer” (e.g., Weber & Herr, 2019). Similarly, it is unclear whether differences in shame would have emerged if the present study was conducted verbally to a clinical psychologist and further research is needed to determine whether online anonymity acts as a buffer against the intensity of shame. In addition, the participants in the current study disclosed a self-selected shame-related event, to maximize ecological validity, however the diversity of experiences they selected for disclosure, as well as how they were disclosed may have confounded findings. Specifically, given the multifaceted nature of shame, the shame-related event for disclosure may not have been the same type of shame experienced by victims of sexual violence. Further studies may benefit from either the inclusion of a measure gauging subjective severity associated with the disclosed event or asking participants to disclose their feelings and thoughts of an experience embedded within the study itself that evokes a type of shame conceptually similar to that faced by victims. Similarly, the present study sought to investigate whether invalidation produces shame, and whether that shame results in an aversion toward further disclosure. Given that all participants were required to re-disclose, and few opted to change the content of their narrative, future designs may benefit from providing an alternative task to re-disclosure, such as a menial cognitive task that has a minimal risk of shame.

Finally, this study investigated the role of invalidation on immediate decisions to re-disclose. However, disclosure of sexual victimization has been argued to be a continuous process, with each successive interaction influencing the decision to re-disclose (Chaudoir & Fisher, 2010; McElvaney et al., 2012). The longitudinal effect of invalidating feedback on re-disclosure choices remains to be explored, as one study has demonstrated changes in measures of distress and arousal the day following feedback (Lepore et al., 2004).

### Summary

The act of disclosure and the disclosure recipient have a synergistic effect in facilitating or hindering recovery. The present study contrasted the role of validating and invalidating feedback upon disclosure of a personal shame-related event, finding that individual perceptions of invalidation is associated with shame, fueling decisions around whether to amend their original narrative, and whether to approach or avoid the original disclosure recipient. As such, this study provides empirical support for the notion that an aversion to being shamed features in individual judgments of further disclosure.
